# Multimodal Image Analysis in Acquired Vitelliform Lesions and Adult-Onset Foveomacular Vitelliform Dystrophy

**DOI:** 10.1155/2016/6037537

**Published:** 2016-04-12

**Authors:** Ricardo Rocha Bastos, Carla Sofia Ferreira, Elisete Brandão, Fernando Falcão-Reis, Ângela M. Carneiro

**Affiliations:** ^1^Department of Ophthalmology, Hospital São João, Porto, Portugal; ^2^Department of Sense Organs, Faculty of Medicine, University of Porto, Portugal

## Abstract

*Purpose*. To characterize vitelliform lesions (VLs) in adult-onset foveomacular vitelliform dystrophy (AOFVD) and acquired vitelliform (AVL) patients using multimodal image analysis.* Methods*. Retrospective study of twenty-eight eyes from nineteen patients diagnosed with AVL or AOFVD. They were evaluated by color fundus photographs, fundus autofluorescence (FAF), fluorescein angiography (FA), and spectral-domain optical coherence tomography (SD-OCT).* Results*. Bilateral VLs were associated with AOFVD (*p* = 0.013). Regular and centered VLs were associated with AOFVD (*p* = 0.004 and *p* = 0.016), whereas irregular and noncentered lesions were more frequent in AVL patients. Visual acuity, greatest linear dimension (GLD), lesion height (LH), and pseudohypopyon were similar between groups. Whereas median LH and GLD in AVL group diminished significantly during follow-up (*p* = 0.009 and *p* = 0.001), AOFVD lesions tended to become larger and thicker.* Conclusions*. When consulting a patient presenting a VL with unknown age of onset, familial history, or previous retinal diseases, some aspects of multimodal imaging assessment may lead the ophthalmologist to a correct diagnosis.

## 1. Introduction

Vitelliform lesions (VLs) correspond to an accumulation of yellowish material in the subretinal space. This phenotype is shared by different retinal diseases, with distinct genetic background and etiologies [1, 2]. These lesions may evolve over time and may be classified in the following stages:

- *Stage I* (previtelliform): normal or only subtle RPE changes (tiny, central honeycomb structure centrally), normal vision.
- *Stage II* (vitelliform): classic “egg-yolk” lesion, normal vision or mild vision loss.
- *Stage III* (pseudohypopyon): layering of lipofuscin, normal vision or mild vision loss.
- *Stage IV* (vitelleruptive): breakup of material gives “scrambled egg” appearance; vision may be mildly decreased.
- *Stage V* (atrophic): central RPE and retinal atrophy; vision may range from 20/30 to 20/200.
- *Stage VI* (choroidal neovascularization, CNV): in about 20% of patients; vision often decreased to 20/200 or worse [1, 2].

When detected in younger patients, VLs usually occur in the setting of Best macular dystrophy (Best disease), an autosomal dominant disease associated with a mutation in bestrophin 1 (BEST1) [3, 4].

Adult-onset foveomacular vitelliform dystrophy (AOFVD) is a subtype of macular pattern dystrophies, being associated with mutations in the peripherin 2 (PRPH2) gene, either sporadic or inherited in an autosomal dominant manner [5, 6]. The age of onset is typically between 30 and 50 years, and it is recognized as a pleomorphic disease, with great variability in size, shape, and distribution of the vitelliform material [1, 5].

Acquired vitelliform lesions (AVL) are a different type of VL associated with multiple retinal diseases such as age-related macular degeneration (AMD), cuticular drusen, tractional maculopathies, pseudoxanthoma elasticum, and central serous chorioretinopathy [1, 7].

All the aforementioned diseases share the lack of direct apposition between the photoreceptor outer segments and the retinal pigment epithelium (RPE), which could delay the phagocytosis of shed outer segment photoreceptor tips, and lead to the accumulation of yellowish subretinal vitelliform material [2, 6–9].

Whereas Best disease may be easily distinguished from other VLs by the age of diagnosis or electrophysiological study, the differential diagnosis between AOFVD and AVL may be difficult in patients without familial history of disease or with simultaneous macular diseases.

In this study we characterize the VL in AOFVD and AVL patients using multimodal imaging analysis, exposing similarities and differences that could help in differential diagnosis between the two entities in clinical practice.

## 2. Methods

This is a retrospective study of patients diagnosed with AVL or AOFVD, who were evaluated in the Department of Ophthalmology of Hospital São João, a tertiary health care center, at Porto, between June 2011 and December 2013. The tenets of the Declaration of Helsinki were followed and local Ethics Committee approval was obtained.

Only eyes having previously undergone multimodal imaging analyses (color fundus photographs, fundus autofluorescence (FAF), fluorescein angiography (FA), and spectral-domain optical coherence tomography (SD-OCT)) were included. Color fundus photographs were obtained with TRC-50EX mydriatic camera (TopCon Medical Systems, Tokyo, Japan). Spectral-domain optical coherence tomography, FAF, and FA were performed using Spectralis HRA + OCT system (Heidelberg Engineering, Heidelberg, Germany).

For each patient we recorded age, type and laterality of VL, associated macular lesions, baseline and final best-corrected visual acuity (VA) using ETDRS charts, and follow-up period. Loss of VA was analyzed as a continuous variable for each letter lost. Vitelliform lesions were evaluated for their stage, integrity of external limiting membrane and the assumed inner segment/outer segment junction (classified as present, absent, or disrupted), intraretinal fluid, and hyporeflective area in the subretinal space both at the baseline and at the final consult. The shape of VL was classified as round or irregular and the location was determined by the foveal topographic relationship and symmetry, being categorized as central or eccentric to the fovea.

Quantitative measurements of anatomical features (greatest linear dimension (GLD) and lesion height (LH)) were recorded using the calipers provided by the review software of the Spectralis HRA + OCT, similar to the method used by Freund et al. [2].

Patients were divided into two groups, AOFVD and AVL, according to familial history and the absence of other previous retinal diseases in the former. In both groups, a normal Arden Index was an obligatory finding. Cases with baseline choroidal neovascularization were excluded. During the follow-up, no patient has received any treatment, such as cataract surgery or CNV intravitreal injections.

Statistical calculations were performed using Statistical Package for Social Sciences (version 20.0; SPSS, Inc., Chicago, Illinois, USA). Descriptive analysis was performed for all variables measured. Categoric variables were compared using the chi-square test, and differences for quantitative variables were analyzed by Mann-Whitney* U* or Kruskal-Wallis test. The association between VA and quantitative measures of lesion was analyzed by Kendall's tau test. The chosen level of statistical significance was *p* < 0.05.

## 3. Results

Twenty-eight eyes of nineteen patients were included in this study. Thirteen patients were diagnosed with AVL (16 eyes) and six with AOFVD (12 eyes). The median follow-up was 10 months, being the 25th–75th percentiles [5–29]. The groups were followed up by a similar median (*p* = 0.782)—the AVL group was followed up by a median of 9 [7; 22] months and AOFVD by 11 [5; 28] months.

A summary of patient characteristics in each group is presented in Table 1. In our case series, VLs were more frequent in patients in their eighth decade and a slight female predominance was observed. We found no statistical differences in the demographic characteristics between the two groups.

### 3.1. Fundoscopic Findings

At presentation, the majority of lesions were stage II, with the classic egg-yolk pattern being present in 8 eyes (50%) of AVL patients and in 7 eyes (58.33%) of AOFVD group. This difference did not achieve statistical significance (*p* = 0.461). Pseudohypopyon was observed in 5 patients (31.25%) in AVL group, compared with 2 cases (16.7%) in AOFVD, which also represented a nonsignificant difference between groups (*p* = 0.662). The presence of vitelleruptive stage was also evenly distributed between groups (3 eyes versus 2 eyes, *p* = 0.662). Atrophy was only verified in one AOFVD case.

Unilateral VLs were present only in AVL group. On the other hand, bilateral VLs were found in 3 AVL patients (23.1%) and in 6 AOFVD patients (100%), being significantly associated with the adult-onset disease (*p* = 0.013).

**Figure 1:**
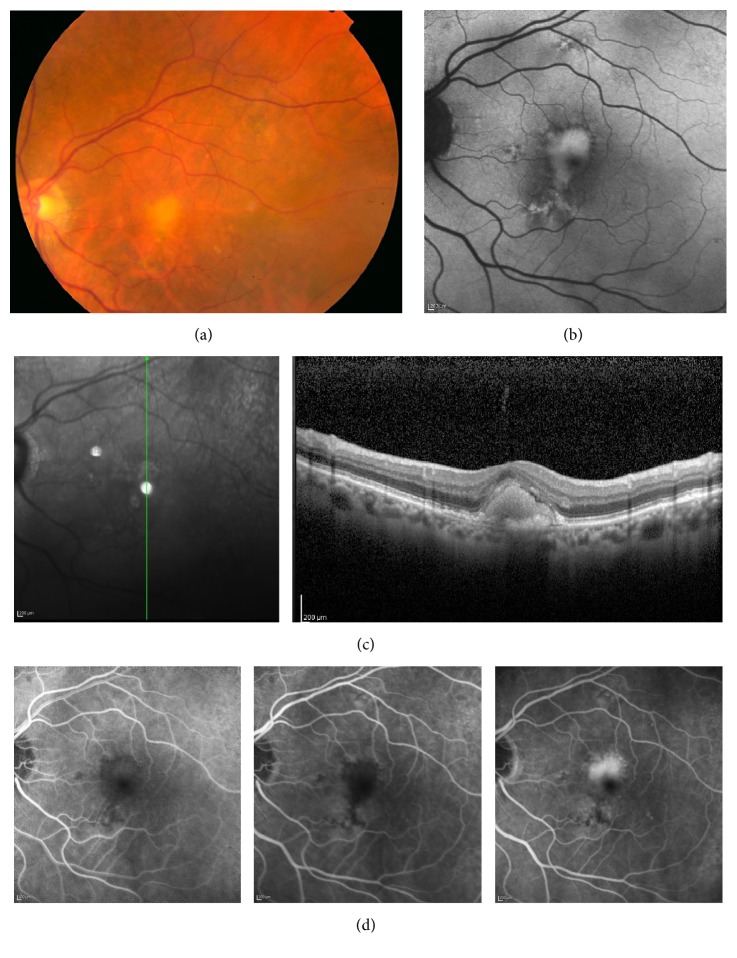
Multimodal imaging of AVL. (a) Color photograph showing an AVL associated with sparse drusen. (b) FAF reveals hyperautofluorescence corresponding to the yellowish material in the color photograph; VL is irregular and in an eccentric position related to the fovea. (c) SD-OCT shows hyperreflective material within the subretinal space. (d) FA revealing blocked fluorescence by material within the subretinal space in the early phases and staining of the AVL during the late phases of angiogram. AVL, acquired vitelliform lesion; FAF, fundus autofluorescence; VL, vitelliform lesion; SD-OCT, spectral-domain optical coherence tomography; FA fluorescein angiography.

**Figure 2:**
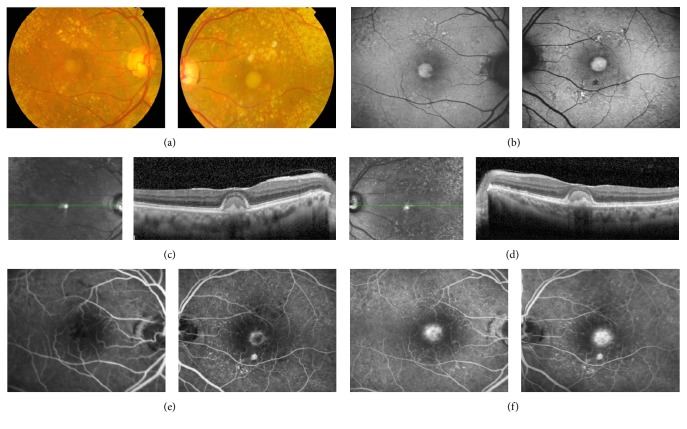
Multimodal imaging of AOFVD. (a) Color photograph showing a VL associated with cuticular drusen. (b) FAF reveals hyperautofluorescence corresponding to the yellowish material in the color photograph; the contours of VL are regular and are centered to the fovea. ((c) and (d)) SD-OCT shows hyperreflective material within the subretinal space in right and left, respectively. (e) FA revealing blocked fluorescence by subretinal material in early phases of angiogram in both eyes, with associated ring of hyperfluorescence in left eye. (f) Staining of VL in the late stages of angiogram. AOFVD, adult-onset foveomacular vitelliform dystrophy; VL, vitelliform lesion; FAF, fundus autofluorescence; SD-OCT, spectral-domain optical coherence tomography; FA, fluorescein angiography.

The shape and location of the VL were significantly different in the two groups. Regular and centered lesions were associated with adult-onset type (*p* = 0.004 and *p* = 0.016, resp.), whereas irregular and eccentric lesions were more frequent in acquired disease (Figures 1 and 2).

### 3.2. OCT Findings

Vitelliform lesions observed in the fundoscopic examination were topographically correspondent to hyperreflective material localized between RPE and Ellipsoid Zone or ELM in SD-OCT (Figures 1 and 2). All clinical and OCT findings in the affected eyes are compared in Table 2.

A disrupted Ellipsoid Zone was statistically associated with adult-onset type of disease both at first and at last clinical evaluation (*p* = 0.020 and *p* = 0.049). In acquired form, either the integrity or the absence of this line was more common than a disruption. There was no relation between the integrity of Ellipsoid Zone and the initial or final VA (*p* = 0.131 and *p* = 0.384, resp.).

Initial median LH was 142.5 *μ*m in AVL patients and 118.5 *μ*m in AOFVD cases. This intergroup difference did not achieve statistical significance (*p* = 0.452). At the last follow-up visit, LH measurements were not significantly different between groups (*p* = 0.291). However, in AVL group, when comparing the initial and final measurement of LH, there was significative shrinkage (142.5 versus 125.5, *p* = 0.009), which was not the case in AOFVD group (118.5 versus 189, *p* = 0.348).

Greatest linear dimension of VL was also similar when we compare the two groups, at both the initial and the last follow-up visit (*p* = 0.336 and *p* = 0.213). Once again, in the AVL group there was shrinkage, with initial and final measurements being statistically different (864 versus 761.5, *p* = 0.001), while in AOFVD group growing was not significant (736 versus 1355, *p* = 0.188).

We were not able to measure VL of 10 eyes in the final observation: 4 patients lost follow-up (6 eyes), 1 developed CNV (1 eye), 2 evolved to vitelli-disruptive-like stage of the disease (2 eyes), and 1 AOFVD case presented an atrophic-like stage of the disease in one eye.

### 3.3. FAF Findings

Vitelliform lesions were hyperautofluorescent in FAF exams (Figures 1 and 2). In some cases with pseudohypopyon, FAF revealed a hypoautofluorescent superior half and a hyperautofluorescent bottom half. Some cases evolved to vitelli-disruptive stage and one AOFVD case presented the atrophic stage of the disease. In either case, residual macular lesions were hypoautofluorescent, revealing damage to the RPE.

### 3.4. Angiographic Findings

In each patient, FA showed early hypofluorescence of the yellowish subretinal VL (Figure 1), occasionally with a halo of hyperfluorescence (Figure 2). A progressive late staining of VL was observed during the angiogram (Figures 1 and 2). Cuticular drusen presented a typical “stars in the sky pattern” in FA. One case developed CNV during the follow-up period.

### 3.5. Additional Findings

Concerning AVL group, early AMD was the most frequent concurrent disease, being found in 7 cases.

We found a correlation between loss of VA and increasing GLD (*p* = 0.034) and a tendency to an increase in thickness (*p* = 0.058).

## 4. Discussion

This study exposed both similarities and differences between AVL and AOFVD patients in a multimodal assessment. Bilateral VLs with regular shape, central location, disrupted Ellipsoid Zone, and the absence of other fundoscopic findings were significantly associated with AOFVD. On the other hand, VA, LH, and GLD did not appear to be distinctive features between these two different clinical entities.

The knowledge about VLs has improved significantly over the last years. In this regard, SD-OCT was determinant to localize vitelliform material in the subretinal space, between RPE and Ellipsoid Zone [3, 8]. Fundus autofluorescence also proved to be useful in the controversial theme regarding the composition of vitelliform material, since typical hyperautofluorescence of VL was consistent with previous histopathological findings of outer segment material, melanin granules, and “lipofuscin-like” material [6, 9]. Fluorescein angiography is a keystone in the diagnosis of retinal disease, and it has been fundamental to identify several distinct entities associated with VL, particularly in AVL patients [2].

It is well known that all VLs share a yellowish appearance in fundoscopic evaluation, a subretinal location and homogeneous hyperreflectivity in SD-OCT, hyperautofluorescence in FAF, and a similar behavior during FA [2, 4, 6, 9, 10]. Theoretically, we can distinguish these patients by the age of diagnosis, familial history, and the presence or absence of associated diseases. However, in clinical practice, this distinction is often difficult since we frequently consult patients for the first time in their sixth to eighth decade, with simultaneous retinal diseases and unknown familial ophthalmological history. In fact, the age of diagnosis in our series was not significantly different between the AVL and AOFVD group. Bilateral disease, although not exclusively, was associated with AOFVD and unilateral disease was present only in the AVL group. This is consistent with a genetic predisposition of AOFVD patients. The association found between AVL and simultaneous retinal diseases, namely, early AMD, was previously reported by several authors and expected according to the diagnostic criteria used for the two groups [2, 6, 9, 11, 12].

We found no statistical difference in VA, LH, and GLD between groups, both at initial and at final observation. However, during the follow-up, we recorded a significant decrease in median LH and GLD in the AVL group. On the other hand, AOFVD lesions became larger and thicker at an apparent larger scale but failed to demonstrate a statistically significant change. This finding could be explained by the different etiologies of these two clinical entities. In AVL patients, VLs are secondary to an underlying retinal disease and could tend to evolve earlier to atrophy. Contrarily, in AOFVD patients VLs occur as a result of a genetic predisposition, becoming atrophic only at later stages. Further studies are necessary to validate this different clinical course between the two entities, namely, the earlier progression to atrophy in AVL patients.

Pseudohypopyon and hyporeflective areas in the subretinal space were not distinctive characteristics between groups. Nevertheless, one must add that pseudohypopyon was observed in 31% of AVL patients, a finding considered to be rare in these patients [2]. A recent report by Gonçalves et al. also presented a case of AVL in a patient with cuticular drusen diagnosed in pseudohypopyon stage [13]. This patient evolved to a stage similar to vitelli-disruptive stage described in Best disease, maintaining a good VA, probably due to a preserved Ellipsoid Zone [1, 2]. In our series, we did not find an association between VA and the integrity of Ellipsoid Zone. Contrasting with previously published series, we found a statistically significant correlation between loss of VA and increasing GLD [2]. These findings could be explained by the size of our sample.

The main distinctive features we found between AVL and AOFVD groups in multimodal imaging were morphological, namely, the shape of VLs and their topographic relationship with the fovea. There was a strong association of centrally located and regular ovoid VL in AOFVD patients, as opposed to irregular shaped and eccentrically located VL in AVL cases. In 1974, Gass first described retinal findings in AOFVD as “round or oval, yellow, subretinal lesions in the foveal area of each eye” [14]. On the contrary, later studies reported significant variability in size and shape of VL both in AOFVD and in AVL patients [1, 2, 5, 7, 15]. The size of our sample could partially explain the statistical significance of our morphological findings. However, since acquired lesions develop from previous retinal diseases, this could define their variable location and shape, leading us to believe that in clinical practice more VLs will be eccentric and irregular in AVL patients when compared to AOFVD.

Our study has several limitations, mostly related to its retrospective nature. The sample size was also small, limiting our ability to detect further statistic correlations or differences. We included both eyes of some patients in the analysis, which may influence the results, though it is partially controlled by the statistical methods used. We measured VL with the caliper function of SD-OCT, which, despite being used in other studies, could introduce some variability in LH and GLD values.

## 5. Conclusions

When consulting a patient presenting a VL with unknown age of onset, familial history, or previous retinal diseases, some aspects of multimodal imaging assessment may lead the ophthalmologist to a correct diagnosis of either AVL or AOFVD. These features may comprise laterality, shape, VL location in relation to fovea, and its size evolution.

## Figures and Tables

**Table 1:** Summary of clinical findings. Results are expressed as follows: ^*∗*^median, 25th–75th percentiles and ^†^absolute number, %. AVL, acquired vitelliform lesions; AOFVD, adult-onset foveomacular vitelliform dystrophy.

Patient characteristics	AVL	AOFVD	*p* value
Age (years)^*∗*^	79 (69–82.50)	73 (61.25–78)	0.210
Male gender^†^	5 (38.5%)	3 (50%)	0.506
Bilateral cases^†^	3 (23.1%)	6 (100%)	0.013
Presence of AMD^†^	7 (53.8%)	0 (0%)	0.044

**Table 2:** Summary of visual acuity and OCT findings. Results are expressed as follows: ^*∗*^median, 25th–75th percentiles and ^†^absolute number, %. AVL, acquired vitelliform lesions; AOFVD, adult-onset foveomacular vitelliform dystrophy; VA, visual acuity; LH, lesion height; GLD, greatest linear dimension; IS/OS, inner segment/outer segment; ELM, external limiting membrane.

	AVL	AOFVD	*p* value
Initial VA (letters)^*∗*^	67.5 (62.25–77)	64.5 (51.25–72.25)	0.347
Final VA (letters)^*∗*^	65.5 (54.75–73)	61 (54.75–73.75)	0.909
Difference in VA (letters)^*∗*^	−4 (−9–4.75)	2 (−19.75–18.50)	0.260
Initial LH (*µ*m)^*∗*^	142.5 (106.5–168)	118.5 (85.75–170.25)	0.452
Final LH (*µ*m)^*∗*^	125.5 (101.75–226.25)	189 (121.25–285.75)	0.291
Difference in LH (*µ*m)^*∗*^	27 (−15–44.5)	12 (1–142.25)	0.892
Initial GLD (*µ*m)^*∗*^	864 (555.25–1505)	736 (487.75–960.75)	0.336
Final GLD (*µ*m)^*∗*^	761.5 (567.5–1128)	1355 (737.75–2033.75)	0.213
Difference in GLD (*µ*m)^*∗*^	33.5 (−3.75–273.50)	163.5 (−31–1238)	0.616
Pseudohypopyon^†^	5 (31.25%)	2 (16.7%)	0.662
Initial interrupted Ellipsoid Zone^†^	4 (25%)	9 (75%)	0.020
Final interrupted Ellipsoid Zone^†^	3 (18.75%)	7 (58.5%)	0.049
Initial integrity of ELM^†^	16 (100%)	12 (100%)	—
Final integrity of ELM^†^	16 (100%)	7 (87.5%)	0.364
Initial hyporeflective subretinal space^†^	9 (56.25%)	8 (66.7%)	0.705
Final hyporeflective subretinal space^†^	9 (64.3%)	4 (50%)	0.662
Regular lesion^†^	4 (25%)	9 (75%)	0.004
Centered lesion	7 (43.75%)	11 (91.7%)	0.016
